# Comparing service delivery models in Finnish dental care: efficiency of the single-visit approach

**DOI:** 10.1186/s12903-025-07111-x

**Published:** 2025-11-28

**Authors:** Märt Vesinurm, Tuomas Nenonen, Tomi Malmström, Paulus Torkki

**Affiliations:** 1 Department of Public Health, University of Helsinki Faculty of Medicine, Helsinki, Finland; 2 Department of Industrial Engineering and Management, Aalto University School of Science, Espoo, Finland; 3 Nordic Healthcare Group, Espoo, Finland

**Keywords:** Dental health services, Integrated delivery systems, Episode of care, Efficiency, Time management

## Abstract

**Objectives:**

This study aimed to investigate the efficiency of two service delivery models in Finnish public dental service (PDS); the treatment plan-based model utilized by two public providers (M1, M2; control units) and the single-visit model (SVM) adopted by a private provider (SV; intervention unit).

**Methods:**

A benchmarking controlled trial design was adopted. Data were collected from electronic health records (EHR) and the study organizations for patient visits, procedures, and resources utilized by the organizations for the entire year of 2013. Data were collected for a total of 156,219 patients and analysis was conducted using descriptive statistics and significance testing.

**Results:**

The intervention unit outperformed the control units on all chosen outcome metrics. The healthcare professionals (HCPs) working in the intervention unit produced on average 84%-102% more, 64%-80% more during a single visit, and 15%-24% more per patient. The episode lengths in the intervention unit were 62%-63% shorter and patients were treated on average with 1.1 visits less than in the control units.

**Conclusions:**

The SVM shows great promise as a service delivery model of choice for routine patients. For a PDS with an obligation to treat the whole patient population, a segmentation based service delivery model could be adopted, which leverages the benefits of the SVM for routine patients and retains the capacity to also treat more complex patients.

## Introduction

Healthcare systems around the world struggle in keeping up with the increasing demand for health services. Dental care is no exception; in fact, dental care is one of the most underutilized sectors of healthcare [1]. Finland is no exception to this trend. The Finnish public dental service (PDS) has long struggled with long waiting times and congestion. In fact, congestion in the PDS has been a challenge for the entire 21 st century [2]. Recent policy changes have further strained the PDS by moving patients’ treatment from the private sector to the PDS [3]. This effect is seen across the board within all socioeconomic and age groups, with some being affected slightly more than others [4]. Results from studies on dental care production in 2016 and 2022, have already observed 3.9% (2016) 3.6% (2022) reduction in all PDS visits [3, 5] despite no signs of reduced overall demand. Simultaneously, the evolving expectations of patients for swift and effective care creates a conundrum for the resource-constrained PDS.

There are many studies showcasing the efficiency of the Finnish PDS. Studies have found that the Finnish PDS has indeed in the past had resources that are uncommitted and could have been reallocated better to improve efficiency, with an estimated 20–30% improvement gap [6, 7]. Resource allocation, with heavy effort going towards frequently examining relatively healthy children at the expense of the adult population has been identified as one key reason for such inefficiencies [8]. Annual or biannual PDS visits are the norm among children, while adults show highly irregular visiting patterns, which is hypothesized to be due to lack of treatment resources [9]. Other hypotheses for the incapability of the Finnish PDS have included tradition, simple lack of resources, failed salary incentives, and inadequate treatment processes [10].

The treatment processes in healthcare service delivery can be organized in many ways. Examples of this include organizing around a specific population (i.e., students, women), urgency of the issue (emergency departments), illnesses and symptoms (i.e., dental clinic, heart center), care practices and processes (i.e., the service delivery models described in this study), or around health outcomes (rehabilitation centers, palliative clinics) [11]. This study focuses on the care practices and processes and specifically around organizing around the demand-supply-based operating modes (DSO) [12]. The DSO framework divides healthcare delivery service processes into seven mutually distinct process types based on the nature of the patient needs (demand) and the professionals needed to meet this demand (supply). These modes are: prevention, emergency, one visit, electives, cure, care, and projects. In the context of routine dental care, the most relevant modes are electives and one visit. Both of these are plannable processes with one key difference: while the one visit is a connected process the electives mode is a disconnected one, requiring more preparation, planning, and scheduling, also implying more handovers and setups (i.e., non-value-adding costs). Previous studies in the context of outpatient clinics have aimed at mathematically modeling better resource allocation and scheduling practices and they have found that in theory, such models would increase the efficiency of the clinics [13, 14]. Additionally, empirical evidence is available from emergency departments, where a service delivery model called ‘fast-track’ or ‘fast-track area’ has been shown to successfully decrease lengths of stay [15, 16]. Empirical evidence is also available in the context of surgeries, where improvements have been found in allocation of surgeons, idle time, and patient satisfaction [17]. In the context of dental care, there is some evidence available on effects of different service delivery models. Prior research in dental care models shows various benefits: the comprehensive care model can improve patient satisfaction [18]; organizational-level factors like assistant-to-dentist ratios and technology use affect efficiency [19]; delivery systems influence dental health disparities [20]; and school-based service delivery models can offer insights into efficient delivery models [21]. In general, however, the evidence of the efficiency of different service delivery models in dental care is scarce.

The objective of this study is quantifying the differences in efficiency between the ‘one visit’ and ‘electives’ operating modes within the context of Finnish dental care. This is achieved by a quasi-experimental benchmarking controlled trial between the ‘one visit’ operating mode-based single-visit model (SVM) of a private provider (SV) and the ‘electives’ operating mode based treatment plan-based service delivery model of two Finnish PDS organizations (M1, M2). A secondary objective of this study was formulating a theoretical PDS delivery model that both functions more effectively based on the mechanisms of action of the SVM but also serves the large variety of the population’s needs as is required of a PDS organization.

.

## Methods

This study adopted a quasi-experimental benchmarking controlled trial [22] design, comparing the efficiency of the intervention unit (SV) utilizing the single-visit model (SVM) to the control units (M1, M2) utilizing the treatment plan-based operating modes. Comprehensive patient-level EHR data from the full year of 2013 was utilized from all three organizations. This study adheres to the STROBE reporting guidelines.

### Service delivery models

The two key differences between the SVM and the treatment plan-based service delivery models are the reduction of handovers and the associated setups, and the more efficient allocation of expensive HCP resources. In comparison to the treatment plan-based model the SVM has fewer handovers, which reduces the number of times the same patient has to be welcomed, cleaned up after, and rescheduled. It also reduces the number of times the HCP has to get reacquainted with the patient’s health status. From the patients perspective this implies less traveling, shorter episode lengths (i.e., time spent in ‘inventory’ or start of care to the point where the health demand is met). The HCP resource is also better allocated as the dentists and dental hygienists are not in the operating room during the handovers and setups.

**Fig. 1:**
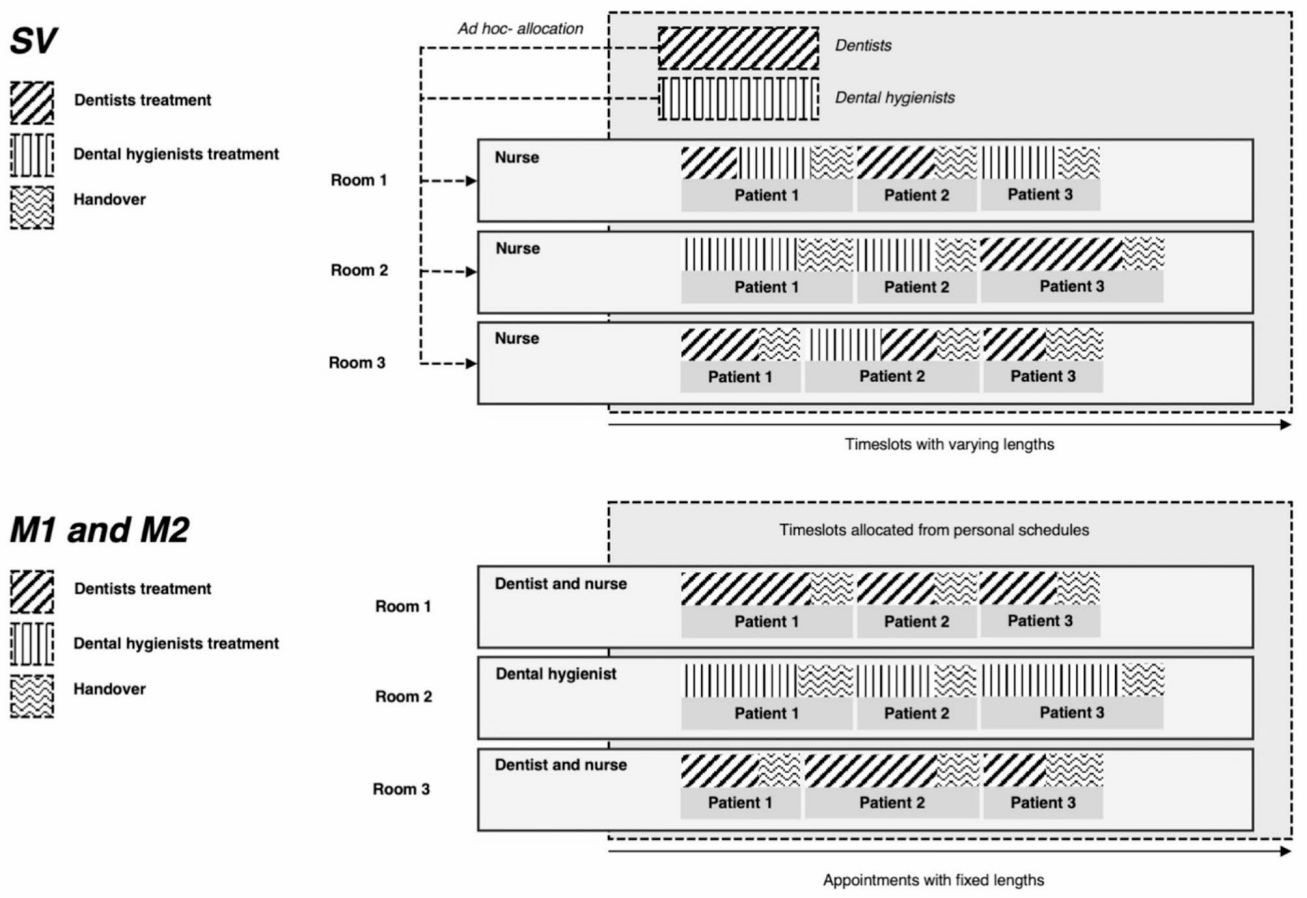
The single-visit service delivery model (upper panel) and the treatment plan-based service delivery model (lower panel) differ in the number of handovers and setups, and in the resource allocation of healthcare professionals

The SVM accepts only routine adult patients. The SVM makes use of flexible appointment times, the movement of dentists and dental hygienists between different operating rooms, and a specialized resource planning system to administrate appointment times. In the SVM resources are organized as follows: each operating room has a dedicated dental nurse, with dentists and dental hygienists being allocated into a shared resource pool. Instead of fixed appointment times, the SVM makes use of flexible appointment times that may be either reduced or increased based on the treatment needs of the patient. Similarly, patients scheduled for appointments are given an hour long window during which their appointment will start (instead of a fixed start time). This allows for previous appointment times to stretch further if needed. The operations are organized as follows: before patients are called in to the operating room the dental nurse is in charge of setting up the operating room and cleaning up after the previous patients appointment. The dental nurse calls in the patient and gets them ready for the dentist, who is called into the room to conduct the dental examination (also referred to as just-in-time (JIT) philosophy in lean management). Based on the status of the patients oral health, the dentists conducts the required procedures at the same sitting (if possible). Conversely, if the dentist deems that only a dental hygienist is needed, one is called into the room and the dentist leaves. After the clinical work is completed, the dentist or dental hygienist leaves the room and the dental nurse completes the handoff of the patient and the setup of the operating room for the next patient. This service delivery model is depicted in Fig. 1, upper panel.

M1 and M2 accept both routine patients and more complex ones. The treatment plan-based service delivery model has fixed appointment lengths, with healthcare professionals (usually) not moving between the operating rooms. The resources of the treatment plan-based model are organized as follows: dentists and dental nurses work in pairs in rooms allocated to them, dental hygienists work alone also in allocated rooms. Appointment times are fixed with a default usually being 30 min to an hour, with personal scheduling calendars. Patients are given fixed appointment start times. The operations are organized as follows: patients are booked for a individual healthcare professional (HCP), who are in charge of setup and cleaning (in the dentist-nurse pair the dentist either participates in this, finalizes the administrative work for the previous patient, or prepares for the next). The HCPs are in the room during the whole appointment length. The process usually starts with an appointment for a dental examination, after which the dentist creates a treatment plan based on the status of the patients oral health. Future appointments are then reserved to (usually the same) dentist and dental hygienist as needed to deliver the care as per the treatment plan. This service delivery model is depicted in Fig. 1, lower panel.

### Study design, measures, and analyses

This study was a benchmarking controlled trial [22] using registry data based on the data collected from the EHRs of SV (intervention unit), M1, and M2 (control units). Outcome measures were descriptively compared between the intervention unit and comparable subpopulations of the control units. The study sample consisted of all patients who visited the intervention and control between 1.1.2013 and 31.12.2013. Children and non-routine patients were excluded. For these patients, data was retrieved on their age, gender, and each individual visit (inc. timestamp, procedures done, HCP resource). In addition, data on the full-time equivalent (FTE) dental nurses, dental hygienists, and dentists were retrieved for each of the organization. The SVM was in use at the intervention unit already before 2013 and as such it is not purely interventional.

As per the guidelines of benchmarking controlled-trials, the patient selection and baseline difference were controlled for between the service delivery models. The patient cohorts of the control units used to calculate outcome measures were limited to routine adult patient (i.e., those treated by the intervention unit). This is achieved by only including adult patients from the control units, who have had procedures done to them that could also be identified from the production mix of the intervention unit. Thus all the compared units were evaluated against their efficiency in treating comparable demand. Efficiency measure differences are only reported between the intervention unit and these subgroups of the control units, M1 (routine) and M2 (routine). From the SV dataset, 86 different procedure codes were identified. The same number for M1 and M2 was 237 and 250 respectively. The majority of SV procedures were dental fillings (SFA-codes) and periodontal procedures (SD-codes). The most common procedure for both M1 and M2 was different dental examinations (SA-codes), after which came dental fillings and surgical procedures (E-codes).

Data on procedures was case mix-adjusted to better reflect the differences in production. This was achieved by using estimates of relative difficulty, which are updated by the Finnish Dental Care Association. Illustratively, a dental filling for one surface (procedure code SFA01) is given the weight 1.00 and other procedures are evaluated in comparison to this. All production was case mix-adjusted (weighted) using these estimates. For each outcome measure, such as weighted output per full-time equivalent (FTE), per patient, or per visit, the weighted output attributable to each individual unit (i.e., each FTE, patient, or visit) was first calculated. These individual-level values were then used to compute aggregate statistics, including means and standard deviations.

The outcome measures were chosen based on the common outcome measures used in healthcare efficiency studies [23] and modified so that the reflected the key differences in production and resource allocation efficiency between the two service delivery models (Table 1). The primary outcome measures were: weighted production per FTE, weighted production per visit, weighted production per patient, visits per patient, and length of episodes. Finally, three descriptive measures were included to further illustrate the differences in the production of the intervention and control units: procedures, weighted procedures, and average procedure weight. Together these measures give a relatively accurate picture of both the inputs and outputs of the different service delivery models. In addition, descriptive statistics are presented on the patient sample and organizations (Table 2 and Table 3).

**Table 1:** Five primary, and three descriptive measures were chosen to describe the relationships between the inputs and outputs of the different service delivery models

Outcome measure	Description
Primary outcome measures	
Weighted production per FTE	How much weighted production is produced per one clinical full-time equivalent?
Weighted production per Visit	How much weighted production is produced per visit?
Weighted production per Patient	How much weighted production is produced per patient?
Visits per Patient	How many separate visits to the dental clinic for each unique patient?
Average episode length (days)	Difference between the first and last visit for the patient.
**Descriptive measures**	
Procedures	How many total procedures produced by the organization?
Weighted production	How much weighted production is produced by the organization?
Average weight of a procedure	What’s the average weight of a procedure done by the organization?

The differences in the outcome measures were analyzed using simple 95%-confidence intervals (CI), which imply statistical significance at the *p* < 0.05 point of a Student’s t-test [24]. Results are reported as 95%-CI’s, as this is preferred over reporting simply *p*-values. Normality of the distributions was assumed based on the central limit theorem (CLT), given the large sample size studied [25].

## Results

### Patient sample and organizational makeup

The patient sample considered totaled 156,219, with M2 being the largest organization at 50%, M2 the second largest at 36%, and SV the smallest at 14% (Table 2). The gender distribution varies slightly between SV, M1, and M2 with a total of 58%, 54%, and 45% women respectively. On average, SV patients were older than those of M1 and M2, at medians (min-max) of 36 (18–113), 28 (0–101), and 24 (0–102). It is expected that a high number of children would reduce the average age in M1 and M2. By sheer volume, SV produced 62% less procedures than M1 and 72.3% less procedures than M2. The total case mix of SV was significantly lower at 86 distinct procedure codes, in comparison to 237 for M1 and 250 for M2. Only considering routine patients for M1 and M2 makes them much more comparable with a total of 21,630 (40%) patients for M1 and 33,188 (44%) for M2 being considered routine, in comparison to 21,976 (100%) for SV. The weight of the average procedure at SV was 24.4% higher than at M1 and 17.6% higher than at M2.

**Table 2:** Demographics of the studied patient population by organization and descriptive measures of the organizations without adjusting for patient mix. Complete patient populations are reported to support the contextualization of the study as these have an inherent effect on the efficiency of the respective organizations. Outcome metrics are reported for subgroups of routine patients

	SV	M1	M2
n, %	21,976 (14)	55,476 (36)	78,767 (50)
Female (n, %)	12,746 (58)	29,957 (54)	35,445 (45)
Age (mean, median, min-max)	39, 36, 18–113	32, 28, 0–101	30, 24, 0–102
Total visits	30,548	137,168	196,459
Procedures	95,034	250,738	343,129
Distinct procedure codes (n)	86	237	250
Average weight of procedure	1,07	0,86	0,91
Proportion of routine patients (n, %)	21,976 (100)	21,630 (40)	33,188 (44)
Proportion of routine patients within adult patients (n, %)	21,976 (100)	21,630 (65)	33,188 (77)
M1 and M2 cover all patient groups (including children and complex cases). In the results, only subgroups of M1 and M2 that had only utilized services (procedures) available at SV were considered. These are noted in Table 4 as M1 (Routine) and M2 (Routine).			

SV employed less FTE’s at 35.7 in comparison to 139.8 at M1 and 220.8 at M2 (Table 3). The proportion of dentists at SV was lower (28%) than in M1 (32%) and M2 (34%). SV employed proportionally more dental hygienists (38%) than M1 (19%) and M2 (15%). Distinctively, 32% of all SV visits were ones that included a dentist and a dental hygienists. M1 and M2 had no visits with such combinations. In addition, SV managed to best utilize their dentists clinical working time, reaching 120% of the theoretical maximum, when the total visit time was tallied for the dentist if they participated with at least one procedure (which explains why the theoretical maximum was exceeded). In comparison, M1 and M2 only reached 64% and 50% respectively. Dental nurses usually do not conduct procedures, which can be expected to have an efficiency lowering effect in the context of this study. The organizational makeups are products of the different delivery models, as staff is hired to according to the needs of each model. Thus, the individual effects of the organizational makeup and distinct delivery model cannot be separated in this analysis.

**Table 3:** The studied organizations have different makeup in terms of healthcare professionals and SV best utilizes the dentist resource for clinical work

	SV	M1	M2
Dental nurses (FTE, %)	12 (34)	68.6 (49)	113.4 (51)
Dental hygienists (FTE, %)	13.6 (38)	27.1 (19)	33.3 (15)
Dentists, (FTE, %)	10.1 (28)	44.1 (32)	74.1 (34)
Total (FTE, %)	35.7 (100)	139.8 (100)	220.8 (100)
Dental nurse visits (n, %)	-	8230 (6)	5894 (3)
Dental hygienist visits (n, %)	3055 (10)	41,150 (30)	41,256 (21)
Dentist visits (n, %)	17,718 (58)	87,788 (64)	147,344 (75)
Dentist + Hygienist visits (n, %)	11,608 (32)	-	-
Total visits (n, %)	30,548 (100)	137,168 (100)	196,459 (100)
Theoretical working time (hours)			
Dentists	17,915	78,536	132,052
Dental hygienists	24,262	48,303	59,343
Actual visit time (hours)			
Dentists	21,537	50,517	66,416
Dental hygienists	13,328	27,815	27,709
Actual/Theoretical (%)			
Dentists	120	64	50
Dental hygienists	55	58	47

Theoretical working time is calculated as 1782 h per full-time equivalent (FTE). SV does not have assigned visits to dentists or hygienists separately, thus the actual visit times for each staff group includes the duration of all visits that included at least one procedure performed by the respective role. Visit time corresponds here to the total time of the patient visit, includes treatment, handovers, and setups

It is important to note that the staffing figures in Table 3 reflect the full organizational workforce and are not limited to the subset of professionals treating routine adult patients. Due to the integrated nature of resource allocation in the comparison units, which serve a wide range of patients, including children and complex cases, it was not feasible to isolate FTEs dedicated solely to routine adult care. As such, Table 3 provides contextual information on the overall resource structure rather than a direct input-output alignment with the outcome measures.

### Outcome measures

In comparing intervention and control units on outcome metrics, only routine patients were considered for the control units. This case mix adjustment procedure is explained in Chap. 2.2. The SV produced 85% and 102% more per FTE than M1 and M2. Similarly, during a single visit, the SV produced 64% and 80% more than the control units respectively. In total, an average of patients of SV received 15% and 24% more treatment than in M1 or M2. The patient’s needs were met with on average 1.1 visits less in SV than in M1 or M2 and the average episode length for SV is 62% shorter than that of M1 and 63% shorter than that of M2. In total production (when only considering routine adult patients) the intervention and control units were comparable, but the average weight of a procedure in SV was 22% and 20% higher than those of M1 and M2.

**Table 4:** Differences between the outcome measures between the intervention and control units within the subpopulations of routine patients

Outcome measure [95%CI]	SV	M1 (Routine)	M2 (Routine)
Primary outcome measures			
Weighted production per FTE	2850 [2835.0–2865.0]	1410 [1337.5–1482.5]	1550 [1543.2–1556.8]
Weighted production per visit	3.6 [3.58–3.62]	2.2 [2.19–2.21]	2.0 [1.99–2.01]
Weighted production per patient	4.6 [4.58–4.62]	4.0 [3.98–4.02]	3.7 [3.68–3.72]
Average visits per patient (median; min-max)	1.4 [1.39–1.41] (1; 1–10)	2.5 [2.48–2.52] (2; 1–21)	2.5 [2.49–2.52] (2; 1–26)
Average episode length (days)	27 [26.14–27.86]	71 [70.20–71.80]	73 [72.32–73.68]
Descriptive measures			
Procedures	95,034	99,134	137,904
Weighted production	101,658	86,949	123,230
Average procedure weight	1.07 [1.07–1.08]	0.88 [0.88–0.89]	0.89 [0.89–0.89]

## Discussion

This study has presented analysis of the differences in the key outcome measures between two different service delivery models: the single-visit model (SVM) used by a private provider (SV, intervention unit) and the treatment plan-based model utilized by two public dental service (PDS) organizations (M1, M2; control units). The intervention unit outperformed control units in every outcome measure when only routine adult patients were considered for the control units, with up to 102% more production per FTE and up to 63% shorter episodes. The organizations were very different in size and makeup, thus, to improve validity of the comparison only subpopulations of routine patients were considered for the control units. A total of 65% (M1) and 77% (M2) of adult patients were considered routine. The study shows that based purely on efficiency, the SVM is superior to the treatment plan-based model. Based on the authors’ knowledge, this is one of the first studies reporting on the effects of this kind of a service delivery model in the context of dental care.

One notable strength of the SVM is its ability to provide complete care during a single visit. This minimizes delays between diagnosis and treatment, which can be particularly beneficial for patients requiring restorative procedures such as fillings. In traditional models, such issues often necessitate a second appointment, potentially prolonging discomfort or pain. By addressing the patient’s needs immediately, the SVM not only improves clinical efficiency but may also enhance the overall patient experience. However, this flexibility comes with trade-offs. The SVM model does not offer fixed appointment start times, which may pose challenges for patients who prefer or require tightly scheduled visits. While the model increases throughput and responsiveness, it may reduce predictability. These differences underscore the importance of aligning service delivery models with the specific needs and preferences of different patient groups.

While this study aims to compare the efficiency of different service delivery models, it is important to acknowledge that the inherent differences between the clinic types, such as population served, complexity of cases, and overall resource allocation strategies may also influence outcomes. The control units serve a broader patient base, including pediatric and complex cases, whereas the intervention unit focuses solely on routine adult patients. This operational context may create systemic differences that cannot be fully disentangled from the service model itself. Thus, this study also has several limitations. First, this study did not consider potential confounders, such as socioeconomic status of the patients, which is known to affect dental care utilization patterns [4, 26–28], and as a private provider, the intervention unit can be assumed to have a more well-off patient population in comparison to the control units. Discussion on the effects of socioeconomic status is somewhat outside the scope of this paper. However, it should be acknowledged that individuals from more affluent backgrounds can afford to purchase more services from private providers. These services may represent a mismatch between health needs and the demand for services—that is, they might not be strictly clinically essential. Conversely, individuals from less affluent backgrounds may utilize dental care services less frequently (demand), despite their oral health condition potentially necessitating treatment (need). A more robust multivariate modeling approach could better control for imbalances in patient demographics and complexity. Future studies could incorporate regression-based adjustment to isolate the effects of service delivery models more precisely. Second, this study did not consider the contextual factors that might affect the success of each of the models. For example, for successful implementation the SVM requires high enough demand, which few places in Finland outside of Helsinki could provide, and private service providers might be incentivized towards higher efficiency regardless of delivery model. In contrast, the control units have to consider and organize around the needs of the whole potential patient population and routine patients cannot be treated in a vacuum. Despite this, successful implementations of SVM have been implemented in Finnish PDS as well [29, 30]. Third, no data was collected on the queue status of any of the organizations that could have been used to study the unmet demand. Fourth, data was not available on the ‘real’ health status of the patients making it impossible to rule out potential overtreatment, which is known to sometimes occur among private providers [31]. Fifth, it is impossible to evaluate the work of the control units’ HCPs separately from their context, thus some of the inefficiencies seen even in the treatment of routine adult patients can be attributed to the fact that these HCPs have to also fit in the rest of the patient population into their schedules. Sixth, the data used in this study is relatively old (2013), however, this does not impact the result reported. Finally, the quality of care, health outcomes, and patient satisfaction were not evaluated.

While there is no available literature on the time allocated for handovers and setups in the dental care context, previous studies have found that managing patient handovers can be estimated to take around 9 min per patient in the context of EDs [32]. Similarly, while most literature focuses on making handovers more efficient through dimensions through better information transfer [33], less focus has been given the fact that it is possible to integrate dental care inside one operation room to almost completely remove handovers as is the case in the SVM. In combination with the JIT approach of ad hoc resource allocation that has been shown to significantly reduce idling time of resources, this study shows a significant boost in efficiency. Further studies are suggested to quantify the separate effects on each of these, by evaluating the precise amount of clinical working time that is saved by first adopting the ad hoc resource allocation, and second, the flexible appointment times. Furthermore, suitability of the SVM and a hybrid service delivery model in different clinical contexts could be explored. Future research should also aim to address potential confounding factors beyond complexity of delivered care. While our study provides descriptive benchmarking between service delivery models, additional analyses using multivariate methods would allow adjustment for patient mix and other contextual differences.

The SVM does not facilitate long care relationships and the patients do not have the possibility of reserving appointments from individual HCPs. Many HCPs believe that long-term care relationships have a key role in providing value-based dental care and improving self-care practices [34]. While this is true for many complex cases, it is prudent to ask if the patients that only seldom require care and even then, the procedures are routine ones, really require a long-term care relationship. An argument could be made that from the patient perspective, access to care is more important than a familiar dentist. It could also be questioned, if long-term care relationships are currently a reality in Finnish PDS at all – in the dataset of this study, the control units’ patients that had over one visit during the year had on average 2.1 different dentists during the year. Only 30% of patients had the same dentist during every visit.

**Fig. 2:**
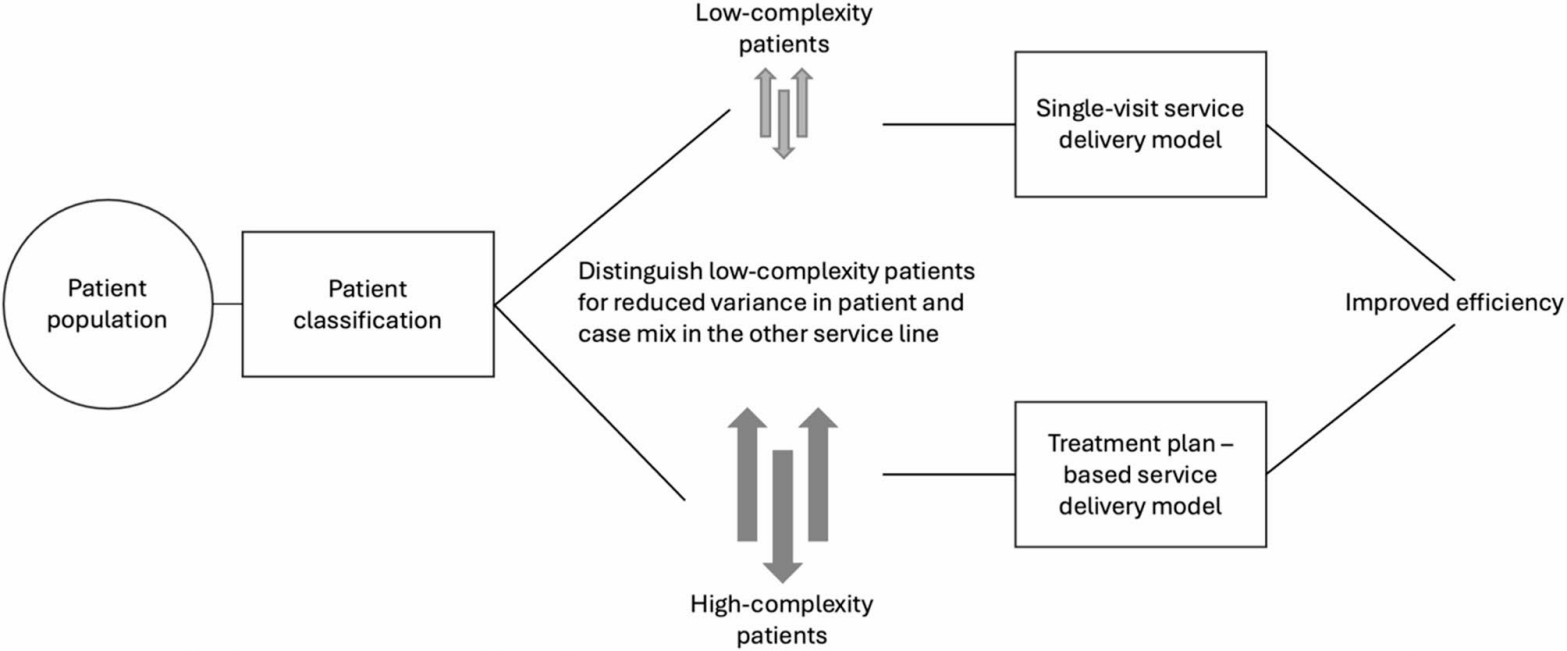
Conceptualization of the segmentation-based dental care delivery model. Patients are segmented into low variation (routine) and high variation (complex) and guided into the corresponding services

A majority of the adult patients in the control units could be thought of as feasible to be treated with the SVM (Table 2). Despite this, a PDS is obliged to provide treatment for all, not just the routine patients. This means that it is conceivable that PDS’ could implement a service delivery system based on two tracks of operating modes. One based on the SVM, and one based on the treatment plan-based model. Patient would them be segmented into low variation (routine) and high variation (complex) and guided into the correct service. Similar proposition have been previously presented in healthcare operations management literature as well [35, 36]. This segmentation-based service delivery model has the potential to greatly improve PDS’ resource allocation and shorten waiting times. Such implementations are already happening around Finland [29, 30]. A conceptual model of such a service delivery model is depicted in Fig. 2. The conceptual model is included as a illustration to situate our findings within the broader DSO framework. The single-visit model studied here aligns closely with the ‘one-visit mode’, characterized by reduced handovers, fewer setups, and more direct resource use. By presenting this conceptual link, we highlight the mechanism through which efficiency gains may arise, while acknowledging that the figure is theoretical rather than derived directly from the empirical data. Thus, the segmentation model should be interpreted as a conceptual framework, rather than a validated implementation model. While this study focuses exclusively on routine adult patients, the figure illustrates one possible pathway for integrating the efficiency benefits of the SVM into broader public dental services.

The implementation of these models in Finnish PDS looks as follows [29, 30]: the target group is adult patients with relatively low care needs. Instead of a set starting time of an appointment, the patients are given an hour long window, during which a text message will be sent to them 30 min before their appointment is set to start. The aim is that as much as possible is done during that one visit (as agreed with the patient), in the limits of what is possible. The appointment starts with an examination. The HCPs move between rooms as they are needed. The listed compatibility criteria are:

1. You are an adult
2. You are flexible with your appointment start time
3. You have the possibility to receive an text message regarding the start time of the appointment.
4. You are able to stay at the clinic for longer than the initially reserved time if needed.

The listed incompatibility criteria are:

1. You are not flexible with your appointment start time
2. You do not own a mobile phone
3. You want to be treated by a specific healthcare professional
4. You have an active care relationship with a specialist dentist

With these rough criteria, these PDS organizations aim to segment the low-complexity patients from the high-complexity ones and guide them into the SVM model. The adoption of such models by the Finnish PDS is in itself an encouraging sign that similar models could prove feasible in other PDS organizations as well and provide a long called for efficiency boost to the dental care production in Finland and elsewhere.

While this study focuses only on routine adult patients, this subgroup represents a substantial portion of the overall patient population in public dental services. Improvements in efficiency for this group alone could yield meaningful system-wide benefits, particularly in reducing waiting times and freeing up resources for more complex cases. Furthermore, any publicly funded healthcare system has to consider and maintain the trust of the majority, despite most of the resources often being channeled towards the complex few.

It is no secret that it is difficult to bring about changes into the way public organizations, including PDS’ are organized, especially without evidence. Our study provides first evidence that the SVM and a segmentation-based service delivery model could increase the efficiency of PDS, representing a promising avenue for managing rising patient demand. Although this study provides positive evidence for the efficiency of the SVM, more research needs to be done on the costs and requirements of implementing such service delivery models in different contexts, the economic benefits of the model, and how such a model fits in with PDS’ production when they still have to take care of the more complex patients as well.

## Data Availability

Due to national legislation, restrictions apply to the availability of clinical data at individual level. For data permission inquiries, please contact the Finnish Social and Health Data Permit Authority (FINDATA) at info@findata.fi.

## Abbreviations

SVM: Single-visit model
PDS: Public dental service
EHR: Electronic health records
M1; M2: Control units
SV: Invervention unit
DSO: Demand-supply-based operating mode
STROBE: Strengthening the reporting of observational studies in epidemiology
HCP: healthcare professional
ED: Emergency department
